# Study on Decarburization and Mechanical Properties of Ultra-Low Carbon Steel by Enlarged Vacuum Chamber Volume

**DOI:** 10.3390/ma18214891

**Published:** 2025-10-25

**Authors:** Kihang Shin, Jimin Yun, Kiwoo Nam, Kwonhoo Kim

**Affiliations:** 1 Department of Metallurgical Engineering, Pukyong National University, 45 Yongso-ro, Nam-gu, Busan 48513, Republic of Korea; kiki64kr@posco.com; 2 Department of Marine Design Convergence Engineering, Pukyong National University, 45 Yongso-ro, Nam-gu, Busan 48513, Republic of Korea; dbswlals333@naver.com; 3 Engineering Research Center, Pukyong National University, 45 Yongso-ro, Nam-gu, Busan 48513, Republic of Korea; namkw@pknu.ac.kr

**Keywords:** ultra-low carbon steel, decarburization, inclusions, mechanical properties, RH degasser

## Abstract

The increasing demand for ultra-low carbon steel (Interstitial free steel of Ti-Nb composite stabilized type) has underscored the importance of the RH degassing process, which is critical to achieving stringent quality standards and high productivity. This study aimed to boost decarburization efficiency by expanding the lower volume of the RH degasser and adjusting the circulation gas flow rates (190 Nm^3^/h, 230 Nm^3^/h, 250 Nm^3^/h). The effects of these variations on decarburization time, carbon content, and mechanical properties were systematically evaluated. The Enlarged RH degasser (ERH) achieved a higher decarburization rate than the conventional RH degasser (CRH) at the same gas flow rate of 190 Nm^3^/h, identifying 230 Nm^3^/h as the optimal rate for ERH. The experimental decarburization times to reach a carbon content of 0.003 wt% in ultra-low carbon steel were 12.4 min for CRH and 10.8 min for ERH, thus reducing the time by 1.6 min. Conversely, the calculated decarburization times were 13.11 min for CRH and 10.75 min for ERH, with ERH showing a reduction of 2.36 min. Consequently, calculated times were 0.76 min longer than experimental times. No significant differences in inclusions were observed between the CRH and ERH at circulation times of 3, 4, and 5 min; however, the mechanical properties of the ERH showed improvements at 4 and 5 min. Therefore, from an economic perspective, 4 min was established as the optimum time. Ultimately, enhancing the lower volume of the RH degasser has increased productivity and decreased production costs.

## 1. Introduction

The research of ultra-low carbon steel with low carbon content has recently increased [1,2,3], and secondary refining and continuous casting processes are important due to strict quality requirements and high productivity goals [4]. The Ruhrstahl Heraeus (RH) degasser stands as the predominant vacuum decarburization unit in secondary refining processes. The RH process treats molten steel through decarburization, deoxidation, and desulfurization under vacuum conditions, and facilitates H and N removal, efficient alloying, rapid homogenization of the ladle steel, and inclusion removal. For enhanced workability, ultra-low carbon steel is refined to diminish the carbon content in molten steel to below 0.003 wt% [5,6,7,8]. This is achieved by initially removing CO gas from blast furnace molten iron, which contains more than 4% carbon, reducing it to 0.03–0.05 wt% using substantial oxygen. Subsequently, the secondary refining RH degassing process eliminates residual oxygen (O) in the molten steel in the form of CO gas, managing the residual carbon (C) in the molten steel to below 0.003 wt%. After the reduction in residual C, the remaining O is extracted from the molten steel as slag (Al_2_O_3_) via a deoxidation process with the addition of Al. However, the resultant Al_2_O_3_ microparticles, approximately 1 μm in size, do not coalesce, impeding their coarsening and making them challenging to remove as slag, thus contributing to the formation of inclusions within the molten steel. Particularly, the Ti alloy added to enhance deep drawability reacts with the Al_2_O_3_ inclusions, leading to the creation of Al-Ti-O complex inclusions [9,10,11].

The mechanism and simulation of the RH decarburization process in ultra-low carbon steel production have been studied [3,12,13,14,15], and numerous studies have examined the decarburization reaction occurring during the steelmaking process [16,17,18,19,20,21]. The decarburization rate was controlled by the mass transfer of either carbon or oxygen, depending on the ratio of carbon content to oxygen concentration [22,23,24]. Kuwabara et al. [25] investigated decarburization behavior by increasing circulation flow rate through the application of argon blowing. The degassing reaction is significantly enhanced by argon gas injection as RH and DH vacuum degassing units increase in size [26,27]. Ti-added Al-deoxidized ultra-low carbon steel formed more complex inclusions than its Ti-free counterpart [28,29]. Additionally, ultra-low carbon steel utilizes O to remove C in the RH process [30,31]. Yet, due to incomplete deoxidation of the molten steel, a significant amount of Al_2_O_3_ forms in the slag until continuous casting; SiO_2_ and MnO inclusions appear during the alloying process [32,33]. Recently, researchers have conducted a few studies on the influence of an argon blowing nozzle layout on the circulating flow rate, and the circulating flow rate increased with an increase in the gas flow rate [34,35,36]. Nozzle clogging was investigated and simulations were performed. The reaction between the nozzle and molten steel was analyzed, and the mechanism of nozzle clogging was studied [37,38,39,40]. The researchers investigated the decarburization behavior of molten steel using a mixed gas and conducted RH model experiments and numerical analyses using snorkels of different diameters [41,42,43,44]. Although numerous studies have addressed decarburization, research on the characteristics of decarburization and the evaluation of mechanical properties due to increased RH degasser vessel volume remains limited.

In this study, the lower volume of the Ruhrstahl–Heraeus (RH) degasser, pivotal in molten steel production, was widened and enhanced to better facilitate gas removal and activate the decarburization reaction in molten steel. The decarburization of the conventional RH degasser (CRH) and the enlarged RH degasser (ERH) was assessed based on the circulation gas flow rate during the production of ultra-low carbon steel (Interstitial free steel of Ti-Nb composite stabilized type of carbon content of 0.003 wt%). Additionally, the mechanical properties of CRH and ERH molten steel were compared.

## 2. Experimental Methods

**Figure 1:**
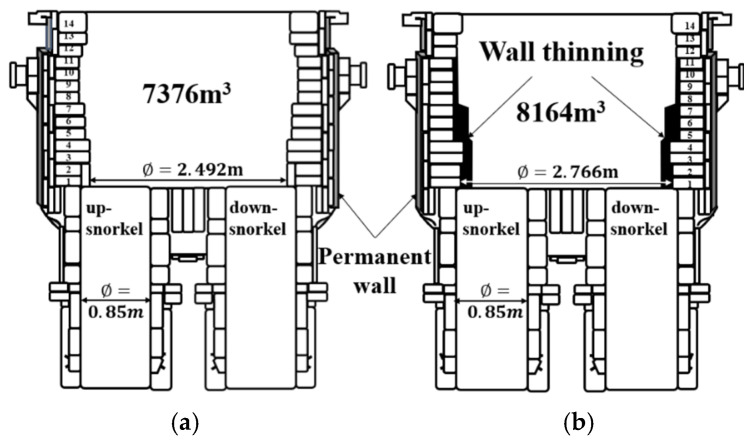
Cross-section of RH degasser vessel. (**a**) CRH, (**b**) ERH.

Figure 1 shows the cross-section of the RH degasser employed in producing ultra-low carbon steel at POSCO’s steelmaking facility. Figure 1a,b portray the Conventional RH degasser (CRH) and the Enlarged RH degasser (ERH), respectively. The modification involved removing the double-walled structure of the ERH, thereby increasing its inner diameter to 2.682 m from 2.492 m in the CRH, a growth of 0.19 m. Correspondingly, the volume expanded from 7376 m^3^ in CRH to 8164 m^3^ in ERH, an increase of 770 m^3^. The thickness of the firebrick lining is 65 mm, with the black area in Figure 1b indicating the expansion. The RH degasser in this study processes 320 t per cycle. The up-snorkel number is 24, and diameter is 0.85 m, respectively. The circulation gas flow rates for CRH are 190 Nm^3^/h, whereas for ERH, they vary at 190, 230, and 250 Nm^3^/h.

The CRH was depressurized from 760 torr to 1 torr and decarburized by injecting 140 Nm^3^/h of circulation gas rate into the up-snorkel until 3 min. After that, 190 Nm^3^/h of circulation gas rate was injected. At approximately 12.4 min, CRH injected deoxidizer Al into the molten steel, subsequently removing it in the form of slag (Al_2_O_3_). After Al deoxidation, if the molten steel temperature and Al wt% are verified at approximately 15 min, the vacuum is reduced to 25–50 torr at approximately 16 min, and the circulation gas flow rate is reduced to 160 Nm^3^/h. Then, Ti, Nb, and Mn for each product are added to match the component range and circulated for 5 min.

The ERH was depressurized from 760 torr to 1 torr and decarburized by injecting 140 Nm^3^/h and 190 Nm^3^/h of circulation gas rate into the up-snorkel until 3 min and 8 min, respectively. After that, 230 Nm^3^/h of circulation gas rate was injected. At approximately 10.8 min, ERH injected deoxidizer Al into the molten steel, and removed it in the form of slag (Al_2_O_3_). After Al deoxidation, if the molten steel temperature and Al wt% are verified at approximately 12 min, the vacuum is reduced to 25–50 torr at approximately 13 min, and the circulation gas flow rate is reduced to 160 Nm^3^/h. Elements such as Ti, Nb, and Mn are then added to reach the specified compositional range and circulated for 3, 4, and 5 min, respectively. The addition amounts for 320 t in CRH and ERH are 0.025 wt% of FerroTi, 0.012 wt% of Nb, and 0.1 wt% of FerroMn, respectively.

**Figure 2:**
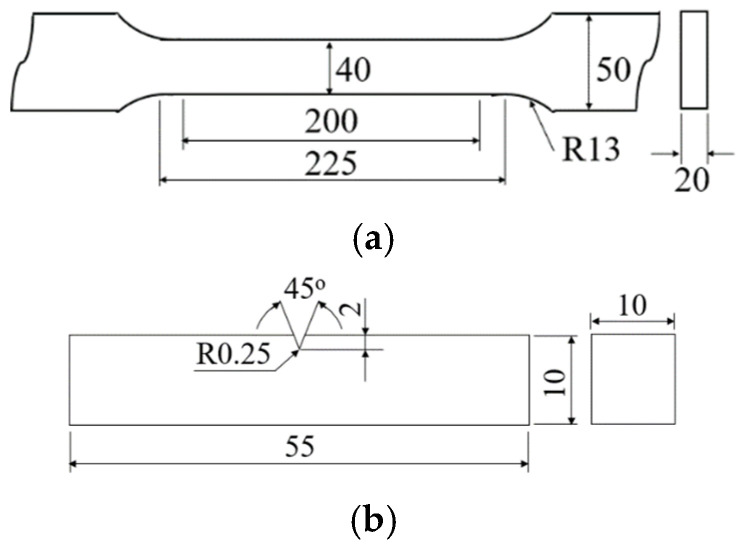
Dimensions and shapes of specimen (unit: mm) (**a**) Tensile, (**b**) Charpy impact.

The circulation time varied from 3 to 5 min for both CRH and ERH. The carbon content in the molten steel was evaluated at 1 min intervals using a carbon and sulfur analyzer (CS844, LECO, St. Joseph, MI, USA). Post-circulation, samples were taken from both CRH and ERH for inclusion analysis. The evaluations occurred 4 times for CRH and 6 times for ERH at each circulation time. All of the inclusions’ size and quantity analyzed using Auto-SEM-EDS (Su-500, Hitachi High-Tech, Hitachi, Japan) on a 3 mm × 3 mm area of mirror-polished specimens. For each specimen, several fields of view were selected to observe inclusions. The average inclusions were measured and then calculated. Three specimens were used, each measured three times and averaged. Tensile properties and impact absorption energy were determined using tensile specimens and V-notch impact specimens. Figure 2a shows the tensile specimen, prepared according to ASTM standard. The length of specimen is 460 mm. Figure 2b presents the standard impact specimen. Tensile and impact specimens were taken from the rolling direction cross-section after hot rolling. The evaluation equipment used a 10 ton universal tensile testing machine (DTU-900MH, Daekyung, Yongin, Republic of Korea) and a 20 kgf-m Charpy impact testing machine (OTC-500, ORIENTAL, Tokyo, Japan).

## 3. Results and Discussion

### 3.1. Carbon Content According to Circulation Gas Flow Rate

The circulation flow rate Q (ton/min) in the RH was determined using Equation (1) [25].

(1) $$\;\;\;\;\;\;\;\;Q=11.4\cdot V^{\frac{1}{3}}\cdot D^{\frac{4}{3}}\cdot \;ln(\frac{P_{0}}{P_{v}}{)}^{\frac{1}{3}}$$

where D is the diameter of the up-snorkel (m), V is the gas flow rate (NL/min), P_0_ is the pressure of the up-snorkel, and P_v_ is the vacuum (torr) within the RH.

The mass transfer coefficient q (m/min) was determined using Equation (2) [45].

(2) $$\;\;\;\;\;\;\;\;\;\;q=0.26\cdot Q^{0.64}\cdot A_{v}\cdot \%C$$

where A_v_ is the cross-sectional area of the RH.

The decarburization rate constant K_c_ (min^−1^) was determined using Equation (3) [23].

(3) $$\;\;\;\;\;\;\;\;\;\;\;\;\;\;\;K_{c}=\frac{Q}{V_{b}\cdot \rho }\cdot \frac{q}{\frac{Q}{\rho }+q}$$

where V_b_ is the volume of steel in the RH, and ρ is the density of the molten steel.

**Figure 3:**
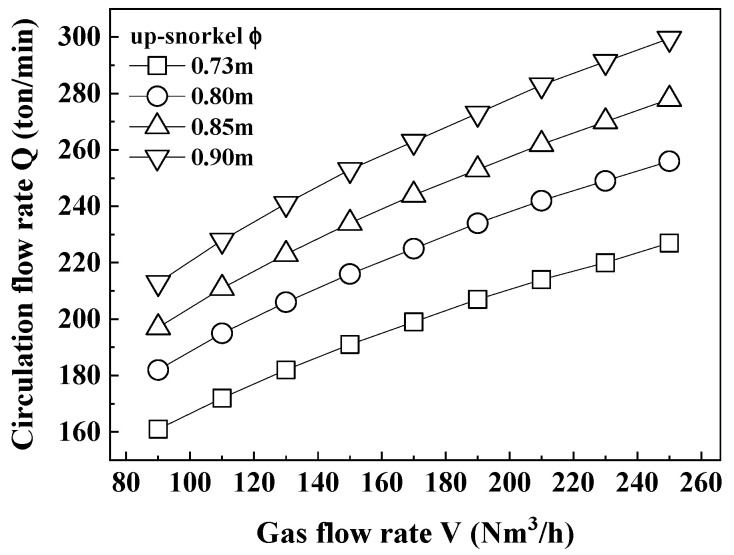
Relationship between circulation flow rate and gas flow rate according to diameter of up-snorkel.

Figure 3 shows the relationship between the circulation flow rate and the gas flow rate, according to the diameter of the up-snorkel obtained from Equation (1). The circulation flow rate was faster as the diameter of the up-snorkel and the circulation gas flow rate increased.

**Figure 4:**
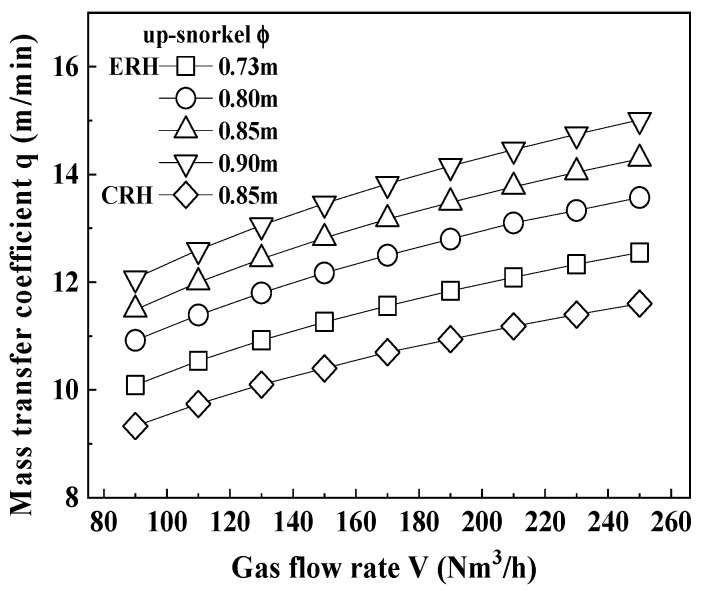
Relationship between mass transfer coefficient and gas flow rate according to up-snorkel diameter.

Figure 4 shows the relationship between the mass transfer coefficient and the gas flow rate according to the diameter of the up-snorkel, which was obtained from Equation (2). The mass transfer coefficient of the ERH was shown to increase as the diameter of the up-snorkel increased. Additionally, for the same diameter of the up-snorkel, the mass transfer coefficient was observed to be larger as the gas flow rate increased.

When comparing the ERH and the CRH, both having the diameter of the up-snorkel of 0.85 m, the mass transfer coefficient of the ERH was found to be greater. Specifically, the mass transfer coefficients for the ERH and CRH were 14.0 m/min and 10.9 m/min, respectively, indicating that the ERH was 3.1 m/min faster.

**Figure 5:**
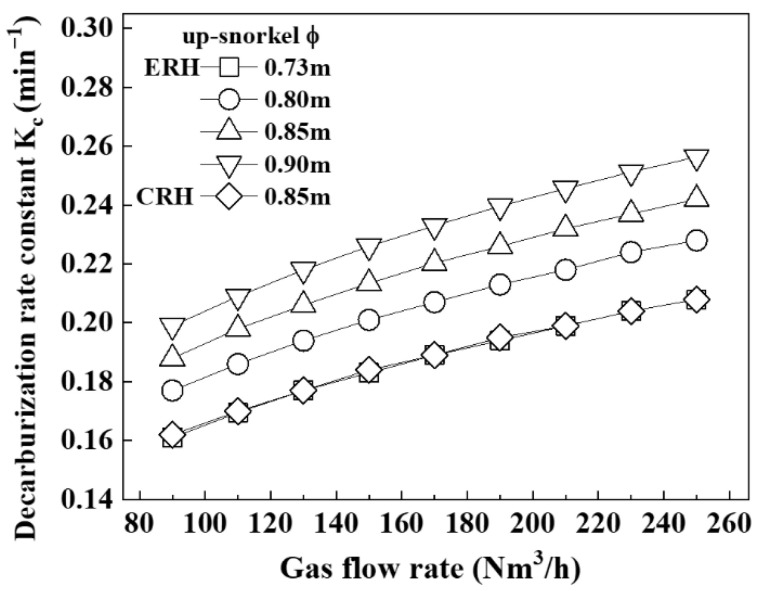
Relationship between decarburization rate constant and gas flow rate according to up-snorkel diameter.

Figure 5 shows the relationship between the decarburization rate constant and the gas flow rate according to the diameter of the up-snorkel, which was obtained from Equation (3). The decarburization rate constant of the ERH was shown to increase as the diameter of the up-snorkel increased. Furthermore, for the same diameter of the up-snorkel, the decarburization rate constant was observed to be greater as the gas flow rate increased. Comparing the ERH and CRH with the diameter of the up-snorkel of 0.85 m, the decarburization rate constant of the ERH was found to be larger. Specifically, the decarburization rate constants for the ERH and CRH were 0.237 min^−1^ and 0.195 min^−1^, respectively, indicating that the ERH was 0.042 min^−1^ greater.

From Figure 3, Figure 4 and Figure 5, as the gas flow rate increased, circulation rate, mass transfer coefficient, and decarburization rate constant increased. However, at a circulation gas flow rate of 250 Nm^3^/h for ERH, the lifespan of the up-snorkel and firebrick was compromised (refer to Figure 4). Thus, the experiment concluded that the optimal circulation gas flow rate for ERH was 230 Nm^3^/h.

Table 1 shows the circulation rate, mass transfer coefficient, and decarburization rate constant according to gas flow rate in the diameter of the up-snorkel of 0.85 m.

**Figure 6:**
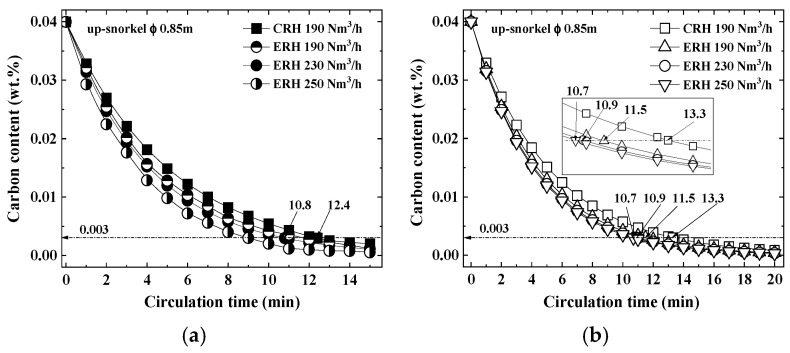
Relationship between carbon content and circulation time according to gas flow rate. (**a**) Experiment, (**b**) Calculation.

Figure 6 shows the correlation between circulation time and carbon content for circulation gas flow rates in CRH and ERH. Figure 6a,b present the experimental results and calculations, respectively. The CRH utilized a circulation gas flow rate of 190 Nm^3^/h, whereas ERH operated at rates of 190, 230, and 250 Nm^3^/h. Decarburization increased with longer circulation times in both CRH and ERH, with ERH exhibiting superior decarburization. This enhanced performance in ERH can be attributed to the enlarged volume of the RH lower vessel, which promoted more vigorous circulation of the 320 ton molten steel batch. Furthermore, even at a circulation gas flow rate of 190 Nm^3^/h, the same as that for CRH, ERH achieved higher decarburization due to its greater mass transfer coefficient and decarburization rate constant. The decarburization in ERH also escalated with increasing circulation gas flow rates.

In Figure 6a, both CRH and ERH processes showed peak decarburization activity at around 9 min for CRH and 8.2 min for ERH, after which the rate of decarburization declined. Nonetheless, no significant variations were observed in mechanical properties or the number of inclusions between the two processes. At this point, the carbon content was 0.00676 wt% for CRH and 0.0056 wt% for ERH. However, since this study aims to produce ultra-low carbon steel with a carbon content of less than or equal to 0.003 wt%, the processes were continued, reaching the target values at roughly 12.4 min for CRH and 10.8 min for ERH; ERH achieved a reduction in circulation time by 1.6 min.

Figure 6b shows the carbon content obtained from the calculations. The circulation gas flow rates were 190 Nm^3^/h for CRH and 190–250 Nm^3^/h for ERH. The relationship between carbon content and time was determined using Equation (4) [46].

C_t_ = C_0_ × exp (−K_c_ × *t*) (4)

where C_0_ denotes the initial carbon content, C_t_ is the carbon content in the melt over time, and *t* represents the decarburization time (in minutes). K_c_ is the decarburization rate constant.

The carbon content, calculated using Equation (4), demonstrated a decline over time, consistent with the trends seen in the experimental results. The duration required to reach the target carbon content of 0.003 wt% for ultra-low carbon steel was identified as 13.3 min for CRH and 10.9 min for ERH, indicating a reduction of 2.4 min with ERH. This outcome exhibited a duration that was 0.8 min longer than the 1.6 min reduction observed in experiment Figure 6a. This is due to the fact that an increase in the inner diameter of the up-snorkel raises the molten steel circulation rate, which causes the decarburization rate constant to continuously increase until reaching a critical value. Beyond this critical value, the molten steel circulation rate no longer influences the decarburization rate constant, which then solely depends on the decarburization reaction coefficient. In case of the inner diameter of the up-snorkel is altered, although the vacuum reaching time is delayed, decarburization time becomes short due to increasing of CO gas production. Hence, increasing the circulation gas flow rate elevates the molten steel circulation rate, enhancing the decarburization reaction. In case of increasing the circulation gas flow rate, decarburization reaction is more effective for the below 0.15 wt% C than above 0.15 wt% C. Initially, above 0.15 wt% C, increasing the flow rate reduces the vacuum capacity which delays the decarburization reaction due to the accumulation of RH degasser exhaust gas. When the carbon content in the molten steel decreases to a certain level or less, the amount of exhaust gas relatively decreases, and increasing the circulation gas flow rate then enhances the decarburization reaction. Thus, it is concluded that increasing the circulation gas flow rate is more efficient for the decarburization reaction when the carbon content in the molten steel is below a certain level than at the initial stage of reaction.

**Figure 7:**
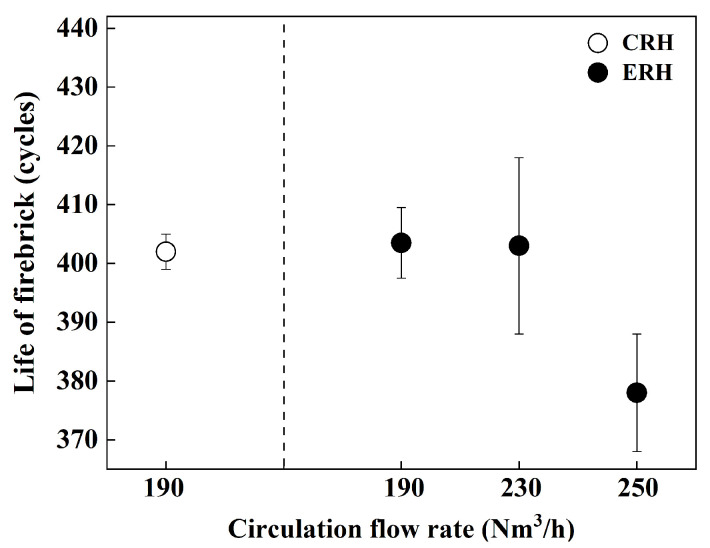
Firebrick lifespan according to gas flow.

Figure 7 shows the lifespan of the RH lower vessel firebrick according to the circulation gas flow rate of CRH and ERH. The lifespan averages five trials each, and I is the standard deviation. The average lifespan of CRH is 403 cycles at a circulation gas flow rate of 190 Nm^3^/h; meanwhile, for ERH it was 402 cycles, 401 cycles, and 378 cycles at circulation gas flow rates of 190 Nm^3^/h, 230 Nm^3^/h, and 250 Nm^3^/h, respectively. Despite high decarburization, at 250 Nm^3^/h the lifespan of the RH lower vessel firebrick is significantly shorter than at lower rates. The lifespans of the firebrick for CRH and ERH are similar at 190 Nm^3^/h and 230 Nm^3^/h. Therefore, considering that the refractory lining exhibited a similar lifetime to that of the CRH at 190 Nm^3^/h, while achieving a more efficient decarburization rate, the ERH at 230 Nm^3^/h was identified as the optimal operating condition.

### 3.2. Inclusions According to Circulation Time

**Figure 8:**
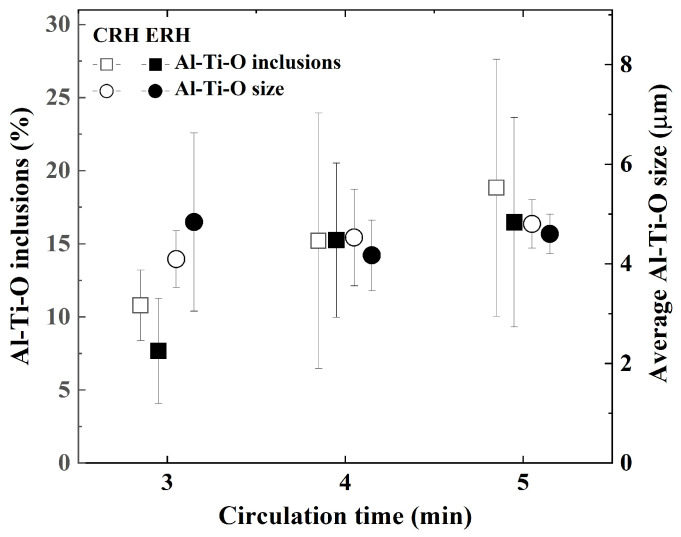
Comparison of Al-Ti-O inclusions and average Al-Ti-O size by circulation time.

**Figure 9:**
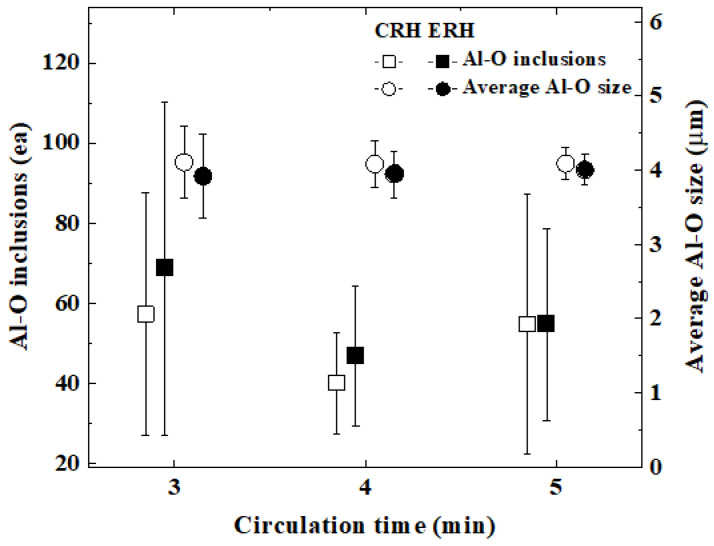
Comparison of Al-O inclusions and average Al-O size by circulation time.

Figure 8 and Figure 9 evaluated the inclusions in ultra-low carbon steel produced by CRH and ERH with circulation gas flow rates of 190 Nm^3^/h and 230 Nm^3^/h, respectively. All of the measured inclusions were targeted. I in each figure indicates the standard deviation.

Figure 8 shows the Al-Ti-O inclusions and the average size of these inclusions for CRH and ERH over circulation times of 3, 4, and 5 min. The Al-Ti-O inclusions in both CRH and ERH increased slightly at 4 and 5 min of the circulation time, and ERH and CRH were almost same at each circulation time. However, Al-Ti-O inclusions exhibited significant deviations for circulation times of 4 and 5 min. The average size of the Al-Ti-O inclusions in CRH increased with the circulation time, whereas it decreased for ERH. Additionally, the ERH was a little small on each circulation time, but the standard deviation was small or similar.

Figure 9 shows the Al-O inclusions and the average size of these inclusions for CRH and ERH across circulation times of 3, 4, and 5 min. Al-O inclusions in ERH and CRH were similar at each circulation time. These inclusions showed a little change as the circulation time increased. However, the average Al-O size in ERH and CRH was nearly the same at each circulation time. The standard deviation was also small. The mechanical properties were subsequently determined at a circulation time of 4 min.

Yamamoto et al. [47] believe that the effect of the elongated Al-Ti-O inclusions larger than 5 μm is stronger than that of the small Al-O inclusions with a size of 5 μm or less on nucleation of voids. Because the elongated Al-Ti-O inclusions with Al-O inclusions larger than 5 μm act as both the voids nucleation sites and the clack propagation paths, they have a great synergistic influence on the local ductility. Consequently, numbers of elongated Al-Ti-O inclusions certainly contribute to the low local ductility.

### 3.3. Mechanical Properties

**Figure 10:**
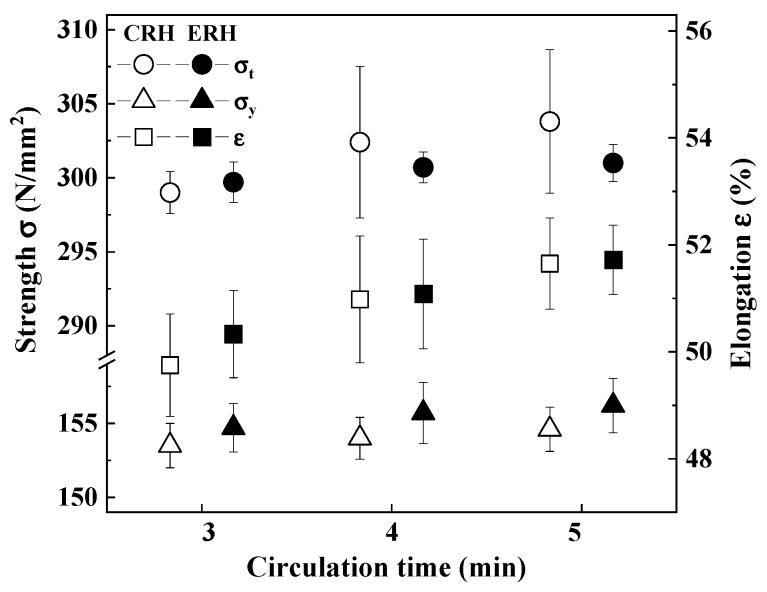
Comparison of mechanical properties by circulation time.

Figure 10 shows the tensile strength (σ_t_), yield strength (σ_y_), and elongation (ε) of CRH and ERH for circulation times of 3, 4, and 5 min. Tensile tests were conducted six times at each circulation time for CRH and ERH. The tensile strength for both CRH and ERH increased slightly with circulation time, with ERH displaying strength similar to or slightly less than CRH at each time. These differences are considered to be within the error margin. The yield strength also increased slightly with circulation time for both CRH and ERH, with ERH showing slightly higher strength than CRH. Furthermore, the elongation for both materials increased as the circulation time increased, with ERH showing marginally greater elongation than CRH at each time. This is believed to be due to the decrease in size and number of inclusions shown in Section 3.2. In other words, as the size and number of inclusions decrease, the initial point of fracture decreases and thus the strength is increased. That is, as the decreasing of inclusions, the number of voids decreases, and voids combined decrease. Yamamoto et al. [47] and Sun et al. [48] found MnS and Al_2_O_3_ inclusions in voids. They judged that stress concentration occurs at the tip of MnS or Al_2_O_3_, which resulted in void initiation. In three mechanical properties, the standard deviation of ERH was small or similar than CRH.

**Figure 11:**
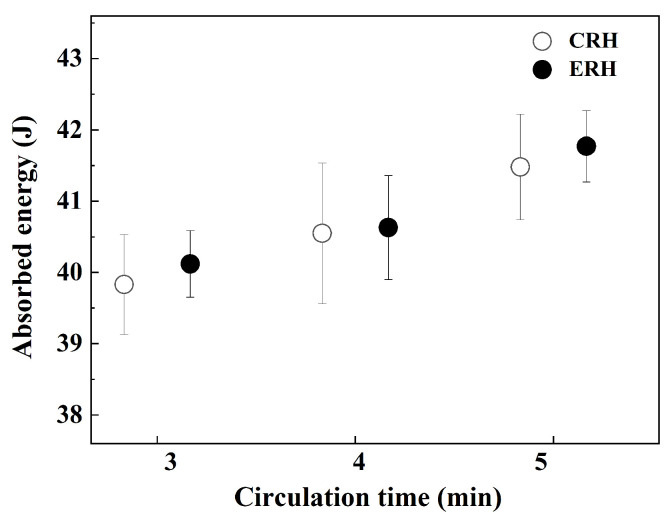
Comparison of Charpy absorbed energy by circulation time.

Figure 11 shows the impact absorption energy for circulation times of 3, 4, and 5 min. Impact tests were conducted six times at each circulation time for CRH and ERH. As the circulation time increased, the impact absorption energy for both CRH and ERH also increased. Although CRH and ERH display similar results, strictly speaking, ERH exhibited slightly higher values. Additionally, the standard deviation for ERH was marginally smaller.

Based on the above results, although the inclusion size and number were observed to be similar between CRH and ERH, the mechanical properties of ERH were assessed to be slightly superior. The expansion of the RH lower volume promotes vigorous molten metal recirculation, which led to a reduced circulation time needed to achieve the ultra-low carbon steel target of 0.003 wt%. Consequently, ERH is considered to have better productivity and economic advantages.

### 3.4. Observation of Inclusions

**Figure 12:**
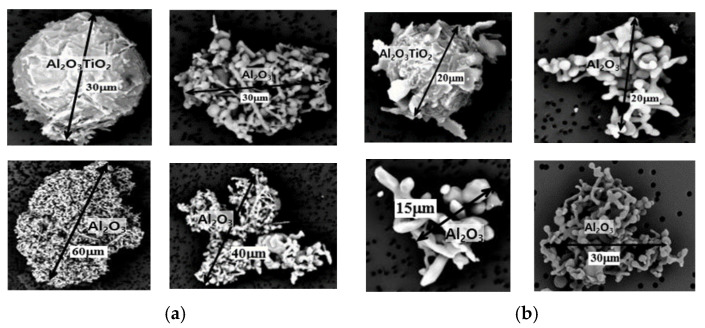
Inclusions in molten steel for circulation time. (**a**) 5 min in CRH, (**b**) 4 min in ERH.

Figure 12a,b shows the agglomerates morphology observed in CRH and ERH. Inclusions at circulation times of 5 min for CRH and 4 min for ERH, respectively, were identified as Al-Ti-O inclusions, with a notably small particle size. These fine inclusions were determined not to significantly influence the mechanical properties, particularly tensile and yield strength [49,50].

**Figure 13:**
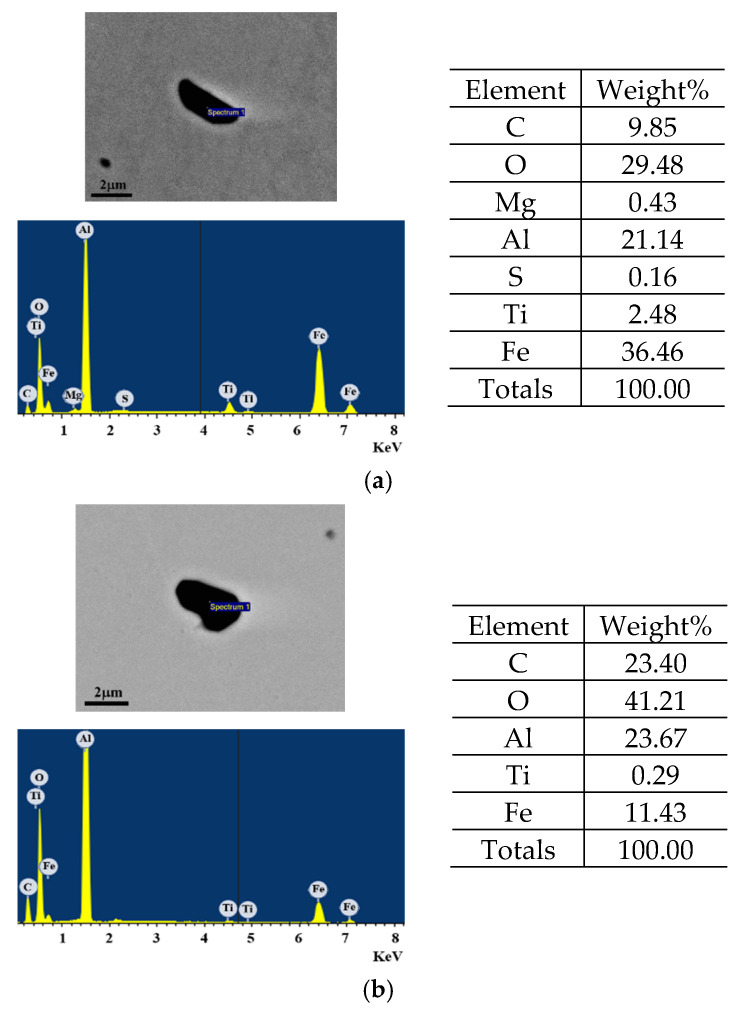
Components analysis of inclusions in molten steel for circulation time. (**a**) 5 min in CRH, (**b**) 4 min in ERH.

Figure 13a,b shows the inclusion composition observed in CRH and ERH. Inclusions of molten steel obtained at circulation times of 5 min for CRH and 4 min for ERH were analyzed. Both CRH Figure 13a and ERH Figure 13b were categorized primarily as Al agglomerates. The key difference between (a) and (b) for Mg and S. (a) contained both Mg and S, while (b) did not contain either.

It was found that the fine inclusions detected in the above did not affect the mechanical strength. Referring to the research of other researcher [47], elongated Al-Ti-O inclusions larger than 5 mm affect the strength, but inclusions smaller than 5 mm had less influence. The inclusions in Figure 8 and Figure 9 are smaller than 5 mm, so they were found to have no effect on strength. The slight increase in strength as the circulation time increased was attributed to the uniform dispersion of fine inclusions.

**Figure 14:**
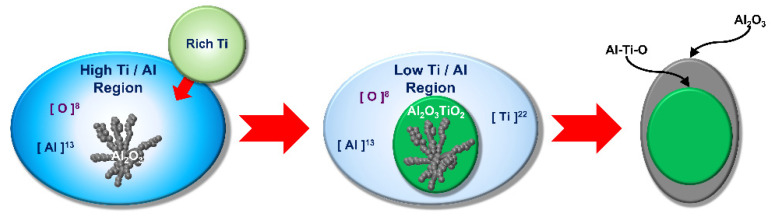
Schematic illustration of formation mechanism of Al-Ti-O inclusions.

Figure 14 schematically shows the inclusions formation process [51]. As the circulation progresses, the Ti content in the oxides decreased. The reduction in Ti is expressed by Equations (5) or (6). As shown in Figure 14, Al-Ti-O surrounded by Al_2_O_3_ appears. They said that an uneven Ti-Al-O core and surface morphology containing small Al_2_O_3_ protrusions exist in some unstable cases. Because of the generation of Ti-oxide and the diffusion of Ti in the melt, a region of low Ti and Al around the inclusion exists. Thus, the generation of Al_2_O_3_ around the Al-Ti-O inclusion is promoted; meanwhile, the transformation of Al-Ti-O inclusion to Al_2_O_3_ would be carried out from the surface to the inner part of the Al-Ti-O inclusion according to the following reactions:

4Al + 3Al_2_TiO_5_ = 3Ti + 5Al_2_O_3_ (5)

2Al + 3Ti*_x_*O = 3*x*Ti + Al_2_O_3_ (6)

### 3.5. Reduce Production Costs

Despite the increased lower volume of the RH degasser and the higher circulation flow rate, the inclusions and mechanical properties of both CRH and ERH were comparable. Consequently, the time required to achieve a carbon content of 0.003 wt% in ultra-low carbon steel for CRH and ERH decreased by 1.6 min, from 12.4 min to 10.8 min. In 2023, the RH degasser utilized in this study processed 1,139,200 tons of ultra-low carbon steel, corresponding to 3560 cycles. The average exchange rate ($1 = 1305 won) for 2023 was applied. The manufacturing cost per minute was $70.5. The 1.6 min reduction in processing time resulted in annual cost savings of $401,557, computed by multiplying 3560 cycles (1,139,200 tons) by $113 (equivalent to $70.5/minute × 1.6 min). The circulation gas (argon) consumption for CRH and ERH was 59 Nm^3^/cycle and 67.5 Nm^3^/cycle, respectively, resulting in an additional consumption of 8.5 Nm^3^/cycle for ERH, totaling an extra 30,260 Nm^3^. Thus, the cost of circulation gas increased by $2418, derived from multiplying 30,260 Nm^3^ by $0.08 (the unit price of argon). A total of $399,138 was saved annually due to the increased lower volume of the RH degasser. After the RH process, the continuous casting operation revealed that CRH and ERH could perform 6 and 10 continuous casts, respectively, for a production of 593.3 casts (1,139,200 tons) for CRH and 356 casts (1,139,200 tons) for ERH. Notably, ERH resulted in 237 fewer casts than CRH. Because of a continuous casting cost of $19,157 per cast, this amounted to savings of $4,540,230, calculated by multiplying 237 casts by $19,157. As a result of the increased lower volume in the RH degasser, the savings from decarburization time and casting costs totaled $4,939,368 by $399,138 and $4,540,230, respectively.

## 4. Conclusions

To enhance the production of ultra-low carbon steel, the RH lower volume was expanded, and the circulation gas flow rates were set to 190 Nm^3^/h, 230 Nm^3^/h, and 250 Nm^3^/h. The impacts on carbon content and mechanical properties were meticulously evaluated. The results obtained are as follows:

(1) Both CRH and ERH showed increased decarburization with prolonged circulation times at the same circulation gas flow rate (190 Nm^3^/h), while ERH exhibited greater decarburization than CRH, especially as the circulation gas flow rate increased. The highest decarburization occurred with a circulation gas flow rate of 250 Nm^3^/h, leading to a reduced lifespan of the up-snorkel and RH lower firebrick. Therefore, the optimal circulation gas flow rate was determined to be 230 Nm^3^/h.
(2) The experimental decarburization times required to reach the 0.003 wt% carbon content in ultra-low carbon steel were 12.4 min for CRH and 10.8 min for ERH, with ERH showing a reduction of 1.6 min. Conversely, the calculated decarburization times were 13.3 min for CRH and 10.9 min for ERH, with ERH achieving a reduction of 2.4 min. As a result, the calculated time was 0.8 min longer than the experimental time.
(3) There were no significant differences in inclusions characteristics between CRH and ERH at circulation times of 3, 4, and 5 min. However, the mechanical properties of ERH proved to be slightly superior at 4 and 5 min, leading to a decision based on economic feasibility. Hence, the expansion of the lower area of the RH degasser was to improve productivity and contribute to a reduction in production costs.

## Figures and Tables

**Table 1:** Various characteristics of CRH and ERH.

	Gas Flow Rate V (Nm^3^/h)	Circulation Flow Rate Q (ton/min)	Mass Transfer Coefficient q (m/min)	Decarburization Rate Constant Kc (min^−1^)
CRH	190	253	10.9	0.195
ERH	190	253	13.5	0.226
	230	270	14.0	0.237
	250	278	14.3	0.242

## Data Availability

The original contributions presented in this study are included in the article. Further inquiries can be directed to the corresponding author.
